# Artificial Intelligence-Powered Mammography: Navigating the Landscape of Deep Learning for Breast Cancer Detection

**DOI:** 10.7759/cureus.56945

**Published:** 2024-03-26

**Authors:** Sahem Al Muhaisen, Omar Safi, Ahmad Ulayan, Sara Aljawamis, Maryam Fakhoury, Haneen Baydoun, Dua Abuquteish

**Affiliations:** 1 Medicine, Faculty of Medicine, The Hashemite University, Zarqa, JOR; 2 Diagnostic Radiology, King Hussein Cancer Center, Amman, JOR; 3 Microbiology, Pathology and Forensic Medicine, Faculty of Medicine, The Hashemite University, Zarqa, JOR; 4 Pathology and Laboratory Medicine, King Hussein Cancer Center, Amman, JOR

**Keywords:** deep-learning, ai and machine learning, mammography, computer-aided diagnosis, artificial intelligence, screening, breast cancer

## Abstract

Worldwide, breast cancer (BC) is one of the most commonly diagnosed malignancies in women. Early detection is key to improving survival rates and health outcomes. This literature review focuses on how artificial intelligence (AI), especially deep learning (DL), can enhance the ability of mammography, a key tool in BC detection, to yield more accurate results. Artificial intelligence has shown promise in reducing diagnostic errors and increasing early cancer detection chances. Nevertheless, significant challenges exist, including the requirement for large amounts of high-quality data and concerns over data privacy.

Despite these hurdles, AI and DL are advancing the field of radiology, offering better ways to diagnose, detect, and treat diseases. The U.S. Food and Drug Administration (FDA) has approved several AI diagnostic tools. Yet, the full potential of these technologies, especially for more advanced screening methods like digital breast tomosynthesis (DBT), depends on further clinical studies and the development of larger databases.

In summary, this review highlights the exciting potential of AI in BC screening. It calls for more research and validation to fully employ the power of AI in clinical practice, ensuring that these technologies can help save lives by improving diagnosis accuracy and efficiency.

## Introduction and background

Machine learning (ML) has become an important aspect of daily clinical practice, especially in radiology. Recent improvements in ML have significantly enhanced diagnostic methods and treatment approaches for many diseases.

Despite the widespread use of computer-aided diagnosis (CAD) in mammography, traditional CAD programs that use prompts to identify potential cancers on mammograms have yet to improve diagnostic precision. Artificial intelligence (AI) has changed due to advances in ML, particularly with deep (multilayered) convolutional neural networks, which have increased the caliber of model predictions. Mammography and digital breast tomosynthesis (DBT) have recently benefited from these deep learning (DL) algorithms. In this review, the authors outline the key technical difficulties and explain how DL functions in the context of DBT and mammography. They then review the state of AI-based clinical applications for mammography, DBT, and radionics and their prospects. Advanced algorithms are available and are on par with radiologists' performance, particularly regarding mammography's ability to detect cancer and predict risk. However, there is still a lack of clinical validation, and how to use DL to improve practice needs to be determined. Digital breast tomosynthesis requires further development of a DL model, which requires the assembly of larger databases. Deep learning will eventually play a significant part in DBT, including creating synthetic images.

This literature review aims to elucidate the function of mammography in detecting breast cancer (BC), including the processing methods employed. It further explores the effectiveness of DL algorithms in contrast to traditional ML and AI methods. Attention is drawn to the flaws and restrictions of AI in breast imaging. Additionally, this paper demonstrates how AI algorithms can be applied to mammography interpretation, highlighting the obstacles that currently hinder practical implementation. Finally, it identifies the challenges and roadblocks in utilizing DL for CAD, providing a comprehensive overview and future directions in this field.

Detecting BC in its early phases is crucial for improved clinical outcomes, and AI has shown promise in increasing specificity and sensitivity in various screening methods, including digital mammography. We believe AI will play a major role in BC screening in the near future.

## Review

Methods

Search Strategy 

A scoping review was undertaken to systematically assess the literature on using AI to detect BC. The method was selected for its ability to handle wide-ranging questions, allowing for the mapping and synthesis of evidence from various studies and sources and facilitating the identification of gaps in the existing body of evidence.

**Figure 1:**
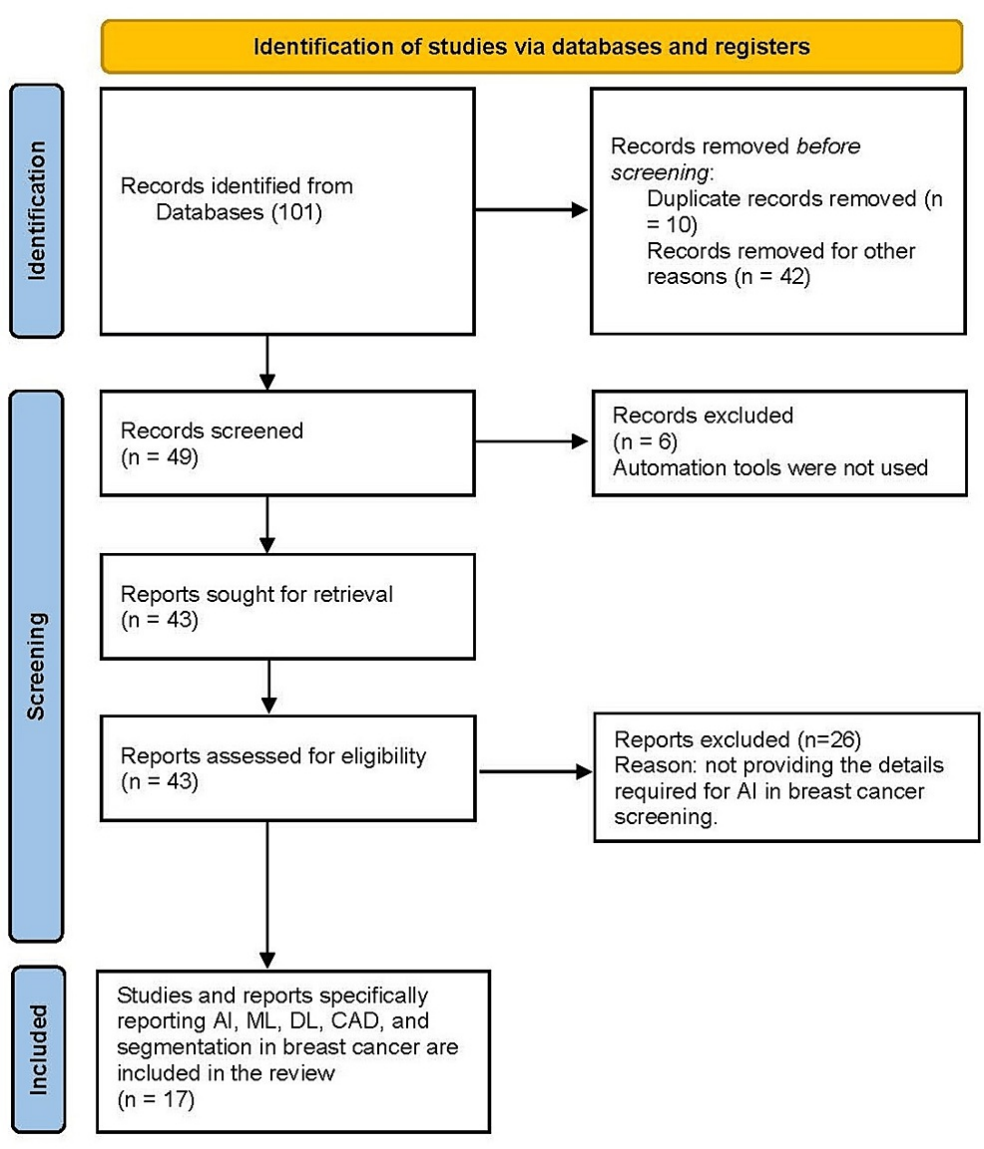
A PRISMA flowchart outlining the study selection process Preferred Reporting Items for Systematic Reviews and Meta-Analyses (PRISMA) 2020 flow diagram for new systematic reviews which included searches of databases and registers only. AI: artificial intelligence; ML: machine learning; DL: deep learning; CAD: computer-aided diagnosis

When developing the scoping review methods, we considered a reporting checklist (an extension of Preferred Reporting Items for Systematic Reviews and Meta-Analyses (PRISMA)) specific to scoping reviews (PRISMA ScR) and a framework and recommendations on review methodology. Keywords such as "artificial intelligence", "computer-aided diagnosis", "breast screening", "mammography", "machine learning", "software", and "breast cancer" were used when searching. We performed a systematic review of studies of AI in radiology published from 2015 to 2022, focusing on mammograms as an example of AI use in radiology. The main search databases were Google Scholar, PubMed, Web of Science, and Scopus. Among the articles identified, non-English language articles, articles published in predatory journals, and commercial software and platforms were excluded. Conventional ML, AI, and DL algorithms were included (Figure 1).

Breast cancer

Breast cancer is among the most common malignancies diagnosed in women worldwide. Many changes in the risk factor profiles and diagnostic modalities for BC have emerged over the past three decades, leading to increases in the prevalence and mortality rates of BC. Currently, people older than 50 make up approximately 80% of BC patients. Both the stage and molecular subtypes of BC affect the survival rate of patients. Breast cancers exhibit heterogeneity in their clinical manifestations, behaviors, and morphology. Breast cancer can be classified into several molecular subtypes based on the level of mRNA gene expression.

The method of choice for identifying BC in asymptomatic women is screening mammography, which has been found to reduce BC-related deaths by 40% to 62% [1]. However, current diagnostic criteria for evaluating mammograms, such as the Breast Imaging Reporting and Data System (BI-RADS), might be constrained by "detection mistakes" (pathology missed) and "interpretation errors" (pathology misinterpreted). Utilizing AI systems that detect irregularities and autonomously classify them as either normal or abnormal is one important way to eliminate errors (interpretation).

This study aimed to examine the most recent advances in the ability of AI to diagnose mammographic images to provide medical imaging experts with a better understanding of how this technology can benefit patients in the future.

In the past few decades, reason has led to human understanding of the world and our role in it since our species' power now lies in our propensity to inquire, research, troubleshoot, and invent. It is believed that only humans possess the capacity to design effective technology, helpful structures, moving works of art, and intelligent narratives.

Artificial intelligence is a highly developed computer program capable of performing activities that typically require human intelligence in areas such as decision-making and visual perception. Deep learning has drastically changed the AI market over the last several years. A subset of ML techniques based on artificial neural networks is known as DL. A subdivision of deep neural networks called convolutional neural networks (CNNs) is extensively used to analyze image data. Like other ML techniques, CNNs rely on the inference of decision boundaries rather than explicit human instructions to enable the computer to carry out specified tasks.

Artificial intelligence in medicine is quickly evolving from the ability to use only hard-coded data to a human-like, rational method of flexible processing of facts and ideas (i.e., history, physical examination, imaging tests). Artificial intelligence is now a creation of human creativity and is in our grasp. Will we have to re-evaluate the importance of human thinking and our role in the universe if AI develops the ability to perceive parts of the world faster, more effectively, with fewer errors, and in ways beyond our capabilities?

It is not new to utilize computers to interpret breast imaging. After receiving approval from the U.S. Food and Drug Administration (FDA) in 1998 and insurance reimbursement in 2002, computer-aided detection (a technique intended to lower the risk of missing pathologies of interest and that aids radiologists in the interpretation of mammograms by marking specific areas of images that might seem atypical) was implemented in mammographic practice in the USA [2]. This adoption took place even though retrospective studies' findings of clinical advantages were not corroborated in actual usage [3].

With the development and promotion of algorithms based on DL, where AI systems learn complex associations in the data that are mathematically described by the use of artificial neural networks, there has been a resurgence in interest in the automated interpretation of mammograms [4]. A computer interprets artificial neural networks while processing hundreds of thousands of photos to learn how to categorize images continually. These networks are layers of mathematical processes that the computer learns on its own and structures so that it can mix and analyze at numerous levels, much like the human brain does. Deep learning and CAD vary primarily in that DL methods do not require explicit programming by a person; instead, individuals learn using their own network until they can accurately identify photos without assistance.

Mammography in breast cancer screening

Mammography is a procedure in which breast tissue is screened for anomalies that could be early signs of BC or other breast conditions. In general, mammography images of interior breast tissues are obtained using modest doses of X-rays [5]. Two plates compress the breasts to generate the image to reduce ray dispersion and obtain a better image without utilizing a high-dose X-ray, where the tissue changes may appear as white zones on a gray contrast [5]. The average mammography time for two images of each breast was approximately 0.4 times the total radiation dosage.

Numerous studies have concentrated on processing digital mammograms to identify the most prevalent signs of cancer: masses or calcifications [6]. Traditionally, experts search for areas that vary from the surrounding tissue in size, shape, contrast, borders, or bright spots. The automation of this process has been suggested using segmentation algorithms [7-9], while some attempts employing neural networks have been made [10-11], with promising outcomes.

Breast Imaging Reporting and Data System

In most countries where a BC screening program is implemented, BI-RADS is now the tool used for communication in mammography reports. BI-RADS was first introduced in the United States in 1995 as a method of standardizing the data collection and quality of mammographic reports [12]. This tool, promoted by the American College of Radiology (ACR), is based on national data that are continually expanded by new cases submitted by licensed physicians [13]. Breast Imaging Reporting and Data System was primarily focused on mammography, but the fourth American edition version, released in 2003, also included ultrasound and magnetic resonance imaging (MRI) screening tools.

Breast imaging studies are assigned one of seven assessment categories, which are as follows: BI-RADS 0: incomplete need for additional imaging evaluation (additional mammographic views or ultrasound) and/or for mammography, obtaining previous images not available at the time of reading; BI-RADS 1: negative, symmetrical, and no masses, architectural distortion, or suspicious calcifications; BI-RADS 2: benign, 0% probability of malignancy; BI-RADS 3: probably benign, <2% probability of malignancy; short interval follow-up suggested; BI-RADS 4: suspicious for malignancy, 2%-94% probability of malignancy for mammography and ultrasound. These can be further divided into BI-RADS 4A: low suspicion for malignancy (2%-9%), BI-RADS 4B: moderate suspicion for malignancy (10%-49%), BI-RADS 4C: high suspicion for malignancy (50%-94%) biopsy, and BI-RADS 5: highly suggestive of malignancy and appropriate action should be taken for patients with a >95% probability of malignancy. BI-RADS 6: known biopsy-proven malignancy. When there are multiple findings, the BI-RADS category for the exam is assigned the highest category in the following hierarchy, from lowest to highest: 1, 2, 3, 6, 0, 4, and 5. The vast majority of screening mammograms fall into BI-RADS 1 or BI-RADS 2. Screening mammograms with suspicious findings should generally be assigned BI-RADS 0 to indicate a call back for diagnostic evaluation, suggesting additional views to confirm and further evaluate the finding.

Major types of breast cancer

The two primary histological subtypes of BC are invasive ductal carcinoma (IDC) and invasive lobular carcinoma (ILC). Invasive lobular carcinomas comprise approximately 10%-15% of all BCs and are distinguished by the discohesive single-file growth of small, spherical tumor cells in the stroma. Mammography and fluorodeoxyglucose (F-18 FDG) PET/CT, two common imaging methods, are less effective at identifying ILCs than identifying IDCs [3]. Compared to IDC, ILC is typically discovered in older patients and at a more advanced stage. Patients with ILC exhibit relatively late recurrence and lower long-term survival than patients with stage- or grade-matched IDC.

E-cadherin (CDH1) protein deficiency occurs in approximately 90% of ILCs [1, 2]. Invasive lobular carcinomas are usually of the luminal A subtype by gene expression profiling, as approximately 90% of ILCs express estrogen receptors more often than IDCs do. Invasive lobular carcinomas also express the progesterone receptor (PR) at high rates (50%-70%), and less than 10% of these cells express HER2/ERBB2 (epidermal growth factor receptor 2). The distinctions between ILC and IDC have recently been characterized at the genetic level [1, 2], but studies of the variations in gene expression have not been as thorough.

Brief description of deep learning

Compared to those of conventional ML and AI, the performance of DL algorithms has significantly improved [14]. The use of DL has increased significantly in several industries, including image classification [12], natural language processing [15], gaming [16], and in particular, the medical imaging industry for the detection and diagnosis of diseases such as skin cancer [17, 18], brain tumor detection, and segmentation [19].

To analyze medical images, many researchers have employed DL methods. The effectiveness of DL mostly depends on the ability of a large number of training samples to learn the descriptive feature mappings of the images, which can produce incredibly accurate classification results. For instance, the network is trained using more than one million photos and more than 1,000 classes of data for the image classification task. However, there is not much training data available in the case of medical photos. Furthermore, collecting a significant number of annotated images is challenging because the annotation process is expensive, and datasets for specific diseases (such as lesions) are rare [20]. As annotations are highly individualized and depend on experts’ skills and knowledge, intraobserver variances are prone to occur when these data samples are annotated in different classes. Numerous research teams have developed various tactics to address the problem of insufficient data, such as trained models with tiny input sizes. The weights are then convolutionalized from the fully connected layers [21], adding some affine transformations (translation, rotation, and flipping [22, 23])) to the data to augment them, and then training the network on the supplemented data, reducing the model parameters and reducing overfitting by employing two-dimensional (2D) patches or three-dimensional (3D) cubes [24, 25] instead of the entire image as input. The last layers are substituted with the new targeted class, and the learning technique is applied using pre-trained weights [26, 27].

Integration of AI in medical imaging

Computer-extracted characteristics can be embedded into ML algorithms, a branch of AI that uses complex statistical approaches to enable robots to perform better at certain tasks by learning data patterns [28]. Furthermore, DL is a subcategory of ML in which many-layered neural networks are utilized to evaluate complicated patterns in inserted data. Deep convolutional neural networks (dCNNs) have been the method of choice for computer visualization and have been employed in several domains of image classification, including breast imaging, since the introduction of the 2012 ImageNet Large Scale Visual Recognition Challenge [29]. When supplied with raw data, dCNNs can develop multiple features that could be connected to a specific result instead of needing human crafting.

Breast masses: identification and classification

Recognizing masses on mammograms can sometimes be challenging, particularly in dense breasts. Several studies have proposed a conjectural fuzzy clustering approach (CrSA-IFCM-NA) based on crow search optimization, in which CrSA-IFCM-NA effectively separates masses from mammogram images, and clusters have been proven to yield good results on the relational index score. The validity of the proposed method results in distinct zone segmentation. In addition, several researchers have created an integrated CAD system that utilizes the You Only Look Once (YOLO) regional DL method, cutting-edge full-resolution deep network (FrCN) models, and dCNNs to recognize crowds of other moving objects. Using the INbreast dataset segmented and categorized into mammograms, the detection accuracy reached 97.9%, assisting radiologists in making precise diagnoses [30-32].

Microcalcifications: identification and classification

Breast calcifications are small calcium salt deposits in the breast tissue that appear as small white patches on mammography. There are two types of calcifications: macrocalcifications and microcalcifications. There are numerous large and coarse macrocalcifications, most of which are normal and age-related. Microcalcifications range in size from 0.1 mm to 1 mm and may or may not have visible masses, maybe early signs of BC [33]. Several CAD techniques have been developed recently. A CNN model was constructed using filtered deep features, completely exploitable by the suggested CNN structure for traditional descriptors, to detect microcalcifications in mammography images [34]. The CNN model performed better at extracting images than handcrafted features. A new approach for distinguishing benign and malignant microcalcifications was devised using an improved Fisher linear discriminant analytical method for the linear transformation of segmented microcalcification data along with a variant of the support vector machine (SVM) to distinguish between the two classes. The average classification accuracy for the 288 regions of interest (ROIs) in the Digital Database for Screening Mammography (DDSM) was 96%, with 139 classified as malignant and 149 as benign [34]. Jian et al. developed a dual-tree, complex wavelet transform-based CAD system for identifying breast microcalcifications [35]. Guo et al. developed a novel hybrid method to detect mammography microcalcifications using a non-linking, simplified pulse-coupled neural network and contourlet transform [36]. An artificial neural network can automatically detect, segment, and categorize masses and microcalcifications during mammography, serving as a resource for radiologists and considerably enhancing their accuracy and productivity.

Segmentation of breast masses

Proper segmentation of masses directly impacts how successfully a patient is treated. Several researchers have used contour maps to segment breast masses on mammograms automatically and assess ROIs obtained from the Mini-Mammographic Image Analysis Society (MIAS) database. The outcomes revealed a mean true positive rate of 91.12% and a precision of 88.08% [37]. Due to the use of low-contrast mammogram images, irregularly shaped masses with speculated edges, and intensity changes in pixels, global segmentation of masses with these features via mammography is very difficult. To segment the breast and suspicious mass areas, several researchers have adopted the mesh-free radial basis function collocation technique. An SVM classifier divided the suspicious areas into normal and abnormal areas. The findings revealed that the sensitivity and specificity of the DDSM dataset were 97.12% and 92.43%, respectively [38]. Plane fitting and dynamic programming were used to identify and categorize breast masses on mammography, considerably improving the accuracy of segmenting breast lesions [39]. Proper segmentation ensures accurate disease categorization and the diagnosis of breast lesions [40]. The application of an automatic picture segmentation technique demonstrated the applicability of DL in medical systems.

Deep learning-based CAD: challenges and obstacles

The development of new deep learning-based CAD systems is challenging. Large databases, which can be expensive to construct, are necessary for DL. Unsupervised learning, where the computer must identify intrinsic factors based on unlabeled pictures, demands high-quality raw data to optimize output; as a result, full-resolution mammographic images, which produce enormous amounts of data files and storage needs, are desired. Although training data requirements have been tailored using methods such as transfer learning and data augmentation [41], validation and testing datasets still need to be more uniform, making it challenging to reproduce published results and contrast algorithms [42]. This is further hindered by the constrained capacity for data sharing and pooling between institutions due to patient privacy requirements imposed by the Health Insurance Portability and Accountability Act (HIPAA), which applies to both imaging and clinical data. The success of the DREAM Challenge demonstrates the value of open science and highlights the significance of collecting sizable, high-quality, anonymous, shareable, and generalizable datasets [43].

Deep learning versus machine learning in mammography

Breast cancer can present in various ways, from overt masses with speculated edges to more subtly asymmetrical or mild microcalcifications, making it challenging to make a precise diagnosis and consistently interpret mammograms. For various recognition tasks, DL outperforms conventional ML methods. Rich feature representations directly learned from massive amounts of data are not constrained by human-designed criteria, which may enable reliable detection of different radiological manifestations of cancer. Another issue with screening mammography is interreader performance variability. Several studies have shown that the sensitivity of detecting BC can range from 74.5% to 92.3% [27]. Deep learning may reduce radiologists' diagnostic performance variability since the software is strongly built on human variation. While viability must be demonstrated in prospective clinical studies, AI is anticipated to aid in mammography-based BC screening by enhancing cancer diagnosis and reducing false-positive recalls.

The results of a 2021 review on the efficacy of AI systems for identifying BC via mammography screening revealed that the quality and quantity of the current evidence are far from sufficient for integrating AI systems into clinical practice. Prior studies aimed to contrast country approaches to gathering evidence, synthesizing it, and formulating policy.

A recent systematic review of AI applications in BC diagnosis concluded that various models, including CNN and the DDSM, were employed to attain timely and precise results. Artificial intelligence demonstrated significant efficacy in analyzing breast cancer images, and CNN was the most frequently utilized algorithm, achieving a notable accuracy rate of 98%. Specificity was the next highest performance metric at 99%. Most of the studies in this review originated in the United States, China, and Japan (Table 1) [44].

**Table 1:** Contributions and findings of various studies related to AI, ML, CAD, and DL This table provides a detailed look at the contributions and findings of various studies related to AI, ML, CAD, and DL in the context of breast cancer screening and diagnosis. CAD: computer-aided diagnosis; DL: deep learning; ML: machine learning; AI: artificial intelligence; YOLO: You Only Look Once regional DL method; CNNs: convolutional neural networks; BC: breast cancer; SVM: support vector machine

Focus area	Technology used	Key findings	References
CAD system implementation	CAD	Improved detection with CAD; CAD received FDA approval and insurance reimbursement despite mixed evidence on clinical advantages and aims to reduce missed pathologies.	[2, 3]
DL vs. Traditional methods	DL	The superior performance of DL, particularly through CNNs, revolutionizes image analysis without requiring explicit programming.	[4]
ML algorithms for image analysis	ML	Effective image classification with ML; ML techniques, including neural networks, demonstrate promise in analyzing digital mammograms for signs of cancer.	[6, 10-11]
AI in diagnostic accuracy	AI	Enhanced diagnostic accuracy with AI; AI systems evolve towards a human-like processing of medical data, showing potential for enhanced diagnostic accuracy.	[28]
DL for microcalcification detection	DL	Accurate detection of microcalcifications with DL; DL excels in the precise detection and classification of microcalcifications, surpassing traditional image analysis methods.	[34]
CAD vs. DL in mammography	CAD and DL	DL offers advantages over traditional CAD. DL offers a significant leap over CAD by learning complex patterns in the data for more accurate mammogram interpretation.	[4, 29]
ML for mass identification	ML	ML is effective in identifying breast masses. ML approaches, like YOLO and CNNs, achieve high accuracy in mass detection, aiding in early and precise breast cancer diagnosis.	[30-32]
AI system validation	AI	The integration of AI in clinical practice demands more extensive validation to ensure reliability and efficacy in BC screening.	[44]
DL algorithms in medical imaging	DL	Significant improvements in disease detection and diagnosis. DL's superior performance in medical imaging underlines its potential for early disease detection and accurate diagnosis.	[14, 17-19]
Segmentation of breast cancer studies	DL and ML	Effective segmentation of breast masses enhances treatment success. Techniques like contour maps and SVM classifiers show high accuracy in segmenting and categorizing breast lesions.	[37-40]

Food and Drug Administration-approved software for breast cancer screening

Because of their potential impact on human health, AI-based diagnostic products have caught the attention of the FDA. The FDA employs the Software as a Medical Device (SaMD) standard to identify software under its regulatory authority. The FDA has cleared nine AI products for BC screening, suspicious lesion identification, and mammogram triage. The 510(k) pathway was predominantly used for clearance, and all clearances were based on retrospective data.

The primary reported outcomes were test performance measures, including sensitivity, specificity, and area under the curve. Notably, tissue biopsy was the gold standard for BC screening accuracy evaluation in most devices. However, other measures of clinical utility, including cancer stage at detection, interval cancer detection, or additional outcomes, were not reported for any of the devices [45-50].

## Conclusions

Deep learning-based systems outperform traditional computer-aided detection systems that use manually created features, coming close to radiologist performance in some tasks. Nevertheless, challenges include the need for large, curated datasets, continuous algorithm updates, and external validation. Recent studies show promising results but lack detailed data descriptions, clarity on the accuracy of various findings, and performance across different demographics and machines. Despite these challenges, AI is expected to play a significant role in the future of mammography and digital breast tomosynthesis assessments, particularly in screening scenarios, suggesting a promising future for AI in enhancing diagnostic accuracy.
